# Taming the neutrophil: Balance between anti‐parasite defence and pathogenesis

**DOI:** 10.1111/pim.12923

**Published:** 2022-05-11

**Authors:** Tara E. Sutherland, Philippe E. Van den Steen

**Affiliations:** ^1^ Faculty of Biology, Medicine and Health, Lydia Becker Institute of Immunology and Inflammation and Wellcome Trust Centre for Cell‐Matrix Research, School of Biological Sciences University of Manchester, Manchester Academic Health Sciences Centre Manchester UK; ^2^ Laboratory of Immunoparasitology, Department of Microbiology, Immunology and Transplantation Rega Institute for Medical Research KU Leuven, University of Leuven Leuven Belgium

The dynamic relationship between the host immune response and an invading parasite is complex and challenging and can sometimes have costly consequences for the host. Well known for their role as first responders to a wide variety of infectious insults, neutrophils are appropriately armed to deal with and help clear invading pathogens through numerous mechanisms such as phagocytosis, degranulation and release of neutrophil extracellular traps (NETs). The production and release of these short‐lived cells by the bone marrow is therefore often potently upregulated in response to infection. However, their defensive roles often come as a trade‐off for the host, with the potential of neutrophils to contribute to disease pathogenesis if responses are not balanced. Here in this special issue of *Parasite Immunology*, a series of reviews explore the versatile range of neutrophil functions during parasite infections, uncovering under‐appreciated roles in defence against a variety of protozoa and helminth organisms. They highlight technical advances to study neutrophil function and pose key questions that will advance our understanding of how neutrophil function can be balanced in order to help control parasite infections while maintaining host health.

Although neutrophils are highly abundant leukocytes in peripheral blood, their study is complicated by a variety of technical issues. This is exemplified by Pollenus et al., who further describe that neutrophils have many‐faceted roles in malaria. Neutrophils contribute to antimalarial immunity through phagocytosis, ROS production and NET formation, but NETs and degranulation may also aggravate malaria complications. Most importantly, malaria modifies the phenotype of neutrophils and may critically curtail their antibacterial function. This phenomenon underpins the increased risk for dangerous coinfections with e.g. *Salmonella*, resulting in potentially lethal bacteraemia.

De Souza‐Viera raises the important notion of neutrophil intrapopulation heterogeneity, in time along the maturation and senescence of these cells, and spatially according to the tissues in which the neutrophils migrate. Differential effects are observed upon infection with *Leishmania*, with infected neutrophils becoming apoptotic and serving as a ‘Trojan horse’ to infect macrophages in a silent mode with activation of anti‐inflammatory pathways. Other neutrophils undergo bystander activation and/or NETosis. This is dependent on a variety of factors, with e.g. increased pentose‐phosphate pathway leading to ROS‐dependent apoptosis. Differential and parasite species‐dependent effects on neutrophil subtypes are also observed. Low‐density granulocytes display increased activation especially with *L. braziliensis*, while *L. donovani* rather induce a regulatory phenotype in neutrophils that contributes to pathology and immune suppression. N2 polarized neutrophils are more permissive to *Leishmania*, while N1 neutrophils mount a stronger inflammatory and parasite‐killing phenotype.

Doolan and Bouchery explore cumulating evidence for a role of NETosis in anti‐hookworm immunity, especially at infective larval stages. While clinical data and in vivo evidence are currently limited, it is clear that neutrophils are capable of releasing NETs in the presence of parasites, leading to reduced worm fitness. Interestingly, part of the immunomodulatory molecules excreted/secreted by helminths prevent host neutrophil recruitment and also potentially allows parasites to evade NETosis. This leads to the suggestion that neutrophils exert a degree of selective pressure on helminths. This review provides thought provoking ideas as to whether targeting early neutrophil mediated immune responses against larval stages of infection could be a potential avenue for development of anti‐hookworm vaccines.

Neutrophil accumulation and NET release have also been observed to occur in the presence of filarial parasites and in particular in response to parasites that harbour the bacterial endosymbiont, *Wolbachia*, as highlighted by Ajendra and Allen. The authors speculate that NETs may be important in tissue‐specific contexts and like with hookworms, NETosis appears an important host immune mechanism to contain larval stages rather than adult parasites. Regardless, as discussed in this review, neutrophils have other diverse host‐protective functions during filarial infection, including production of mediators such as s100a8/9 proteins that may contribute to parasite killing. It is also interesting to speculate that neutrophils may in fact instruct and co‐operate with other immune cells and molecules to help facilitate worm killing. Aside from roles in innate immunity, the authors highlight more recent work to suggest neutrophils may influence induction of protective and reparative type 2 immune responses that are often dominant in later stages of filariasis and hookworm infections.

Sanches et al. review a series of studies that describe the different contributions that neutrophils can make during Schistosomiasis, from releasing cytokines and chemokines at the site of initial infection to influence host‐immunity, to their proposed role during hepatic fibrotic granuloma formation around eggs. Interestingly, neutrophils can have a pro‐inflammatory or tissue reparative phenotype depending on their spatial organization within a granuloma, suggesting divergent functions during *Schistosoma* infections. Utilizing evidence from alternative model infection systems, the authors propose that neutrophil recruitment into the liver during schistosomiasis may be regulated by inflammasomes and that neutrophils themselves could be a key source of inflammasome activated IL‐1 and IL‐18. Further investigation into the interplay between neutrophils and inflammasomes during parasite infection is clearly warranted and could be a promising avenue to understand the balance between immunity and pathology.

Neutrophils are not only key during mammalian parasite infections but also represent a key innate immune defence mechanism in aquatic organisms. Buchmann provides an intriguing and expert review on the ancient origin of neutrophil‐like responses that occur following pathogen invasions of aquatic vertebrate and invertebrate species. Much like mammalian counterparts, a subpopulation of neutrophils can be mobilized to the site of infection where they can recognize PAMPs and engage a range of mechanisms including NET formation, phagocytosis and degranulation in order to prevent spread of infection. However, not surprisingly, aquatic pathogens have also evolved numerous and sophisticated mechanisms to evade host neutrophilic responses.

Taken together, this series of timely reviews shines a light on the multitude of functions neutrophils play during parasite infections. Parasites have evolved evasive strategies to limit neutrophil functions, highlighting the important evolutionary role these cells play in host defence. There is still a long way to go before we have a clear molecular understanding of how neutrophils engage with parasites, co‐ordinate other innate immune cells, influence adaptive immune responses or conversely mediate tissue damage and pathology. Further studies are also needed to better understand the complex relationship between neutrophil heterogeneity and their functional response to a variety of parasites. Improved experimental models, clinical studies, and technologies should pave the way for answering questions that may allow for new therapies that specifically take advantage of neutrophil functions in host‐defence but limit any detrimental outcomes on host pathology.

### PEER REVIEW

The peer review history for this article is available at https://publons.com/publon/10.1111/pim.12923.

